# The Efficacy of Oral Vitamin Supplementation in Dry Eye Disease: A Systematic Review

**DOI:** 10.1007/s44402-026-00079-3

**Published:** 2026-04-15

**Authors:** Hamidreza Heidari, Maria Markoulli, Jayashree Arcot, Asgar Doostdar, Azadeh Tavakoli

**Affiliations:** 1 School of Optometry and Vision Science, Faculty of Medicine, UNSW Sydney, Kensington, New South Wales Australia; 2 Food and Health group, School of Chemical Engineering, Faculty of Engineering, UNSW Sydney, Kensington, New South Wales Australia; 3 Department of Optometry, School of Rehabilitation Sciences, Iran University of Medical Sciences, Tehran, Iran

**Keywords:** Dry eye disease, Ocular surface disease, Vitamin A, Vitamin B12, Vitamin C, Vitamin D

## Abstract

**Introduction:**

Vitamins are micronutrients with a wide range of anti-inflammatory, immunomodulatory, regulatory and antioxidant properties. Several studies have shown that vitamins play a crucial role in the homoeostasis of the ocular surface. Deficiency of certain vitamins might be associated with the severity and incidence of dry eye disease.

**Method:**

This paper reviewed the current evidence on the effect of oral vitamin supplementation on signs and symptoms of dry eye disease. The literature review was conducted in five English archive databases (Pubmed, Cochrane, Embase Ovid, Web of Science and Scopus) from April 2024 to January 2025, following the Preferred Reporting Items for Systematic Reviews and Meta-Analyses (PRISMA) guidelines (Prospero CRD42024629589). Eligibility criteria included studies that reported the Schirmer test, tear film break-up time, corneal fluorescein staining scores, lid hyperaemia, the ocular surface disease index and visual analogue pain scale.

**Results:**

A total of 13 eligible studies, which included vitamins A, B1, B12 and D, were assessed by two independent reviewers for quality and data extraction. Of these, most investigated the efficacy of vitamin D (61.5%), vitamin A (23%) and a mix of vitamin B1 and mecobalamin (15.5%). No studies reported on the efficacy of vitamin C supplementation, and none were judged to be of high quality.

**Conclusion:**

The available evidence is limited due to not being well-controlled or adequately powered, having a very short follow-up duration and variable dosage of supplementation.

Key Points

- Among all vitamins, vitamin D is the most frequently studied in relation to dry eye disease.
- No studies have evaluated the efficacy of vitamin C supplementation as a sole intervention on signs and symptoms of dry eye disease.
- Vitamin B12 has not been investigated separately for its potential role in dry eye disease treatment.

## Introduction

Dry eye disease is characterised by loss of homoeostasis of the tear film and impacts the lives of a vast number of individuals worldwide. Dry eye disease affects about 12% of the world’s population, has a higher prevalence in Africa (47.9%) than in North America (4.6%) and is reported to be among the most frequent reasons for patient visits to eye care practitioners worldwide [1]. The condition is characterised by a vicious cycle of tear film instability and hyperosmolarity, which increases ocular surface inflammation, damage and neurosensory abnormalities [1]. Additionally, significant ocular discomfort, difficulties in daily functioning, diminished vision, poor general health, anxiety and depression are associated with moderate to severe dry eye disease [2]. Increasing severity of dry eye disease is also associated with corneal thinning [3], limbal corneal epithelial cell dysfunction [4], ulceration [5], perforation [6] and even corneal scarring [7]. The commonly reported ocular discomfort symptoms in dry eye disease are dryness, redness, foreign body sensation, heaviness, pain, light sensitivity, discharge, itching and eye strain [2, 8].

The management of dry eye disease relies on restoring the altered homoeostasis of the ocular surface and thereby preventing this vicious cycle [9, 10]. Artificial tears are the mainstay of therapy and are a preferred first-line treatment option due to their lower side effect profile and non-invasive character, thereby providing temporary symptom relief [9, 11]. Corticosteroids are used for both short and long-term management of inflammation, but carry the risk of side effects, including a higher risk of cataract formation and increased intraocular pressure [12], as well as increasing the risk of opportunistic ocular infections [13]. Despite the availability of current treatments, they mostly provide only temporary relief, leaving patients searching for more sustainable solutions. Although dry eye disease is a multifactorial condition without a fully elucidated pathogenesis, recent reports have highlighted the role of immunologic and inflammatory processes [14, 15], with a growing body of evidence suggesting that dry eye disease can also be an autoimmune disease [16, 17].

The role of nutrition in the prevention and treatment of dry eye disease has been undervalued [18]. Poor nutrition is the second-highest risk factor for death and disability-adjusted life years globally, contributing to 10.3 million deaths and 229.1 million disability-adjusted life-years (DALY) [19]. A report of the Tear Film and Ocular Surface Society (TFOS) Lifestyle series on nutrition has shown that dietary essential nutrients, including vitamins, play an important role in metabolic functions, reducing oxidative stress and systemic immune regulation of the body, as well as the ocular surface [18]. Some studies have established a link between vitamin deficiencies, especially vitamins A and C, and ocular surface compromise [20–22]. Typically, vitamins A, B12, C and D are considered to be most effective in addressing ocular surface diseases [23]. Both vitamins A and C are naturally present in the human tear film and ocular surface for epithelial cell differentiation, metabolism and regeneration of other antioxidants [24–26]. While vitamins D and B12 have anti-inflammatory and immunomodulatory properties, they also have the capability to modulate corneal wound healing, thereby enhancing the function of the corneal epithelial barrier and reinnervation in the maintenance of ocular health [27–30]. The association between vitamin deficiencies and dry eye disease, particularly conjunctival squamous metaplasia [31] and a loss of conjunctival goblet cells [32, 33], has been noted previously, and systemic supplementation has been effective in individuals with low vitamin intake [34–43]. The growing evidence points to the viability of dry eye disease management using a nutritional approach based on the anti-oxidative, anti-inflammatory and immunomodulatory actions of vitamins to preserve ocular surface homoeostasis.

This systematic review aims to investigate the role of oral vitamin supplementation in the management of dry eye disease, critically narrowing its scope to the evidence for vitamins A, B12, C and D. The review addresses gaps in the literature and assesses the methodological quality of the available studies, in order to provide consolidated research-based evidence to clinicians and researchers on the role of oral vitamin supplementation. It includes information about how dietary supplements could enhance both objective and subjective outcomes in relation to their therapeutic effect in the treatment of dry eye disease. Objective findings, including Schirmer test scores, tear film break-up time (TBUT), corneal fluorescein staining scores (CFSS) and lid hyperaemia were considered as primary outcomes. These signs are considered reliable indicators of tear production, ocular surface health quality and improvement in the progression of the disease. Subjective outcomes comprised secondary outcome measures, including the ocular surface disease index (OSDI) and visual analogue pain scale (VAS).

## Methods

This systematic review adhered to the guidelines set by the Preferred Reporting Items for Systematic Reviews and Meta-Analyses (PRISMA) and was registered with Prospero (Prospero CRD42024629589) [44]; no amendments were performed after the registry. Only articles written in English were considered. Six English electronic databases were searched (Pubmed, Cochrane, Embase Ovid, Web of Science, Google Scholar and Scopus) to find studies evaluating the efficacy of oral supplementation with vitamins on dry eye disease published up to *11 January 2025*. Citations retrieved from the electronic databases were collated into an EndNote library (https://endnote.com/?srsltid=AfmBOorPCnlwMuK7ADkDtEYxcUgL075HyCmzTT1bZnCCnPMFDBjaKXjB) [45]. After the removal of duplicates, the library was imported into Covidence (Covidence systematic review software, Veritas Health Innovation, covidence.org). Title and abstract screening were done separately by two reviewers (HH, AD), with the relevant articles being chosen for full-text review. Conflicts of interest at any stage of the process were discussed among reviewers to reach agreement, or, if needed, resolved through a third investigator (MM). The following primary search terms were used: “Vitamins”, “Dry Eye Disease” and “Effect”; the specific search terms are available in *Supplementary Material* *1*. No limitations in study type, age group, disease severity or geographical restrictions were imposed in the search strategy.

### Inclusion and Exclusion Criteria

Articles were included if they:

(I) were written in English.
(II) were categorised as either a randomised controlled trial or a prospective, cross-sectional or case control study which evaluated the impact of oral supplementation in patients with dry eye disease, compared with healthy controls, regardless of age, gender or race; observational studies including cohort, case-control, interventional and cross-sectional studies on dry eye disease and healthy controls, encompassing both prospective and retrospective designs.
(III) reported on the dosage, concentration or frequency of oral administration of the vitamins.
(IV) performed at least one of the following tests: Schirmer (SH), TBUT, CFSS, lid hyperaemia, OSDI or VAS.
(V) Followed the participants for at least 3 days after the initiation of supplement therapy.

Reports excluded were grey literature articles without sufficient detail, reviews, case reports, laboratory studies, letters, commentaries or conference abstracts. Additionally, studies that discussed dry eye disease secondary to systemic diseases or other ocular surface disorders (such as graft-versus-host disease, Stevens–Johnson syndrome, ocular mucous membrane pemphigoid or anti-glaucoma medications) were excluded, as were any studies that included dry eye disease and other simultaneous ocular diseases, incomplete scientific articles and papers not written in the English language.

### Data Extraction

Extracted data included the following information: first author’s name, year of publication, study period, treatment in the comparison/control group, type of vitamin intervention, study country/region, study design, number of participants, type of oral vitamin intake, follow-up period, vitamin dosage and frequency of usage. Data for each follow-up time point after treatment for the same group were extracted as independent outcomes (Table 1). For articles with missing data or where means and standard deviations were provided graphically, an email was sent twice to the corresponding authors as a follow-up; however, none responded or supplied additional data. Therefore, means and standard deviations provided in graphical form were extracted using the WebPlotDigitizer website (automeris.io/WebPlotDigitizer).

**Table 1:** Baseline characteristics of included studies.

First author	Year	Vitamin type	Treatment in the comparison group	Study period (year.month)	Location	Type of study	Follow-up duration (days)	Sample size (*N*)	Type of oral vitamin intake	Vitamin dose	Frequency
Najjaran [62]	2022	Vit D	Artificial Tears	2019.11–2022.01	Iran	RCT	56	50	Pearl	50,000 IU	Weekly
Lin [50]	2022	Vit D	–	2019.01–2019.12	China	RCT	30, 90	45	Soft capsule	2000 IU	Once daily
El Said [61]	2021	Vit D	–	2020.03–2020.12	Egypt	Non-RCT (case-control)	14, 42, 70	39	–	The dose for supplementation was calculated related to the serum VD level	Once daily
Yang [43]	2017	Vit D	–	2015.03–2015.11	Australia	Non-RCT (case-control)	60	25	Capsule	1000 IU	Once daily
Hwang [58]	2018	Vit D	Artificial tears	2015.06–2016.06	South Korea	Non-RCT (case-control)	14	9	–	2000 IU	Once daily
Karaca [55]	2018	Vit D	–	Not mentioned	Turkey	Non-RCT (cross-sectional)	56, 84, 168	40	–	50,000 until 56 days then 1500–2000 until 168 days	Weekly
Fayed [63]	2022	Vit D	–	2020.03–2021.03	Egypt	Non-RCT	106	42^a^	–	2000 IU	Once daily
Kanwal [59]	2024	Vit D	Artificial tears	2021.09–2022.07	Pakistan	Non-RCT (case-control)	15, 30, 60, 90	54	–	6000 IU	Once daily
Ren [51]	2020	Vit B1 & Mec*	Artificial Tears/Corticosteroid eye drops	2018.10–2019.03	China	RCT	30,60	40, 32^b^	Tablet	Not mentioned	Not mentioned
Ren [52]	2022	Vit B1 & Mec*	Artificial Tears	2018.11–2021.03	China	RCT	30,90	94	Tablet	Not mentioned	Three times a day
Gupta [49]	2021	Vit A	Omega 3	2019.02–2019.08	India	Randomised Prospective comparative	30, 90, 180	35	Capsule	Not mentioned	Once daily
Alanazi [54]	2019	Vit A	–	Not mentioned	Saudi Arabia	Non-RCT	3	30^c^	Tablet	5000 IU	Once daily
Jadeja [60]	2023	Vit A	–	2019.06–2021.06	India	Non-RCT	30,90^d^	42	Capsule	200,000 IU	Once daily

*IU* International Unit, Mec* Mecobalamin, *RCT* randomised clinical trial, *Vit* Vitamin.

^a^Sample size was composed of 12 patients with vitamin D deficiency (VDD) and 30 patients win non-VDD.

^b^40 participants were recruited in the oral vitamin B1, mecobalamin and artificial tears group and 32 patients were enroled in oral vitamin B1, mecobalamin, corticosteroid eye drops and artificial tears.

^c^Only male participants.

^d^Patients supplemented at 0, 1, 14 and 28 days, then visited for follow-up after 1 and 3 months.

### Quality Assessment

For each study, two reviewers (HH, AD) assessed the methodological quality and risk of bias of either randomised clinical trials (RCTs) or case-control studies independently using the Revised Cochrane risk-of-bias tool for randomised trials (RoB 2) [46] or Newcastle–Ottawa Quality Assessment Scale (NOS) adapted for Cross-Sectional Studies and Case-Control Studies [47]. RoB 2 assessment of bias classifies the overall results into three different scores: low risk, some concerns and high risk of bias. Additionally, the RoB 2 main domains include: risk of bias arising from the randomisation process, deviations from the intended interventions, effect of assignment to intervention and effect of adhering to intervention, missing outcome, measurement of the outcome and selection of the reported result. For case-control and cross-sectional studies, the NOS score categorises the quality assessment of results into three levels, i.e., good, fair and poor quality. The NOS is based on an assessment, as shown in Table 2 for both types of studies included in this review. Any discrepancies raised during quality assessment by independent reviewers were resolved through discussion or adjudication with all authors.

**Table 2:** Newcastle–Ottawa scale for interventional studies.

Study ID	Type of vitamin	Year	Selection				Comparability	Exposure			Total score
Case control studies			Adequate definition of cases	Representativeness of the cases	Selection of controls	Definition of controls	Control for important factor	Ascertainment of exposure	Same method of ascertainment for cases and controls	Non-response rate	
Kanwal [59]	Vit D	2024	**–**	⋆	⋆	**–**	⋆	**–**	**–**	**–**	3
El Said [61]	Vit D	2021	**–**	⋆	⋆	**–**	⋆	**–**	**–**	**–**	3
Hwang [58]	Vit D	2018	**–**	**–**	⋆	**–**	⋆	**–**	**–**	⋆	3
Yang [43]	Vit D	2017	**–**	**–**	⋆	**–**	⋆	**–**	**–**	⋆	3
Fayed [63]	Vit D	2022	**–**	**–**	⋆	**–**	⋆	**–**	**–**	⋆	3
Alanazi [54]	Vit A	2019	**–**	**–**	⋆	⋆	⋆	**–**	**–**	⋆	4
**Cross-sectional studies**			**Representativeness of the sample**	**Sample size**	**Non-respondents**	**Ascertainment of the exposure**	**Comparability**	**Assessment of the outcome**	**Statistical test**		
Jadeja [60]	Vit A	2023	**–**	⋆	**–**	**–**	⋆	**–**	**–**		2
Karaca [55]	Vit D	2018	**–**	⋆	**–**	**–**	⋆	⋆	⋆		4

A higher overall score corresponds to a lower risk of bias; a score of five or less (out of nine) indicates a high risk of bias. Each ⋆ equals 1 point.

*Vit* vitamin.

## Results

### Literature retrieval results

**Fig. 1:**
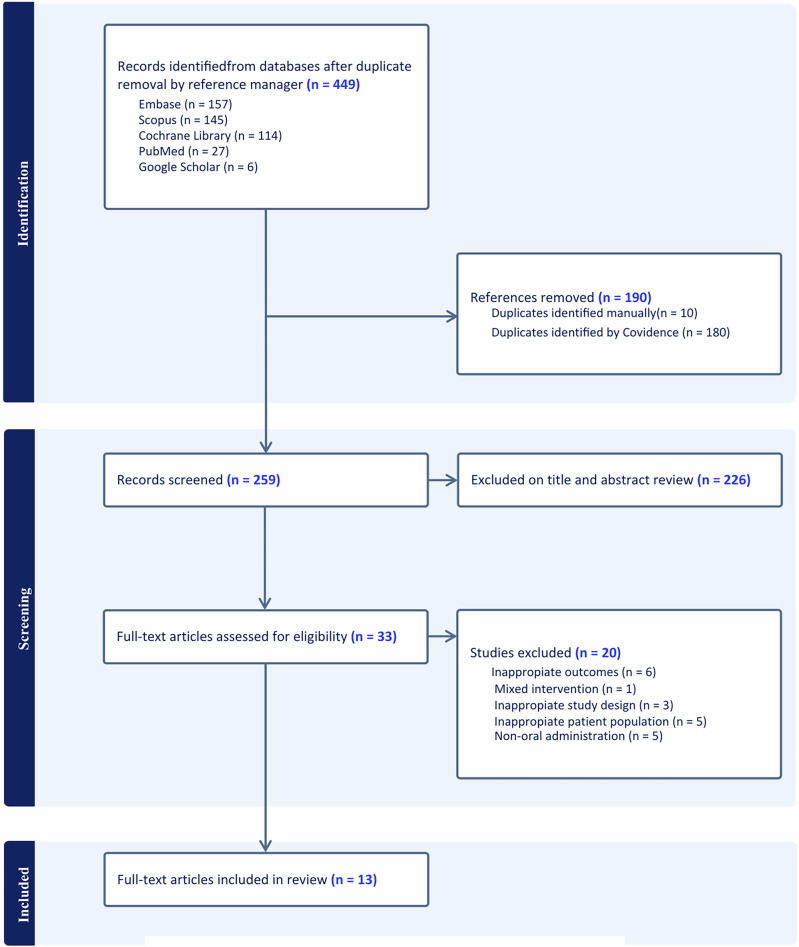
Preferred Reporting Items for Systematic Reviews and Meta-Analyses (PRISMA) flow diagram for identifying articles for inclusion.

Initially, all of the retrieved studies from the five databases were imported into EndNote reference manager software to remove duplicates. This resulted in 488 articles being imported into Covidence. Subsequently, 190 duplicates were removed and 258 articles were excluded based on their title and abstract (Fig. 1). The full text and data of the remaining 33 studies, judged as “eligible” or “potentially eligible”, were evaluated [42, 43, 48–78]. Of these, 13 articles had at least one outcome for SH, TBUT, CFSS, lid hyperaemia, OSDI or VAS [43, 48–51, 53, 54, 57–62]. Twenty studies [42, 52, 55, 56, 63–78] were excluded due to inappropriate outcome measures, mixed intervention, non-oral route of administration, incomplete information or unrelated design and population of study in the full text review. A total of 13 screened studies that met the pre-specified eligibility criteria were included for final review [43, 48–51, 53, 54, 57–64] (see Fig. 1 and Table 1).

### Characteristics of Included Studies

All 13 studies were full-text articles [43, 48–51, 53, 54, 57–62]; the key characteristics of these studies are summarised in Table 1. Most of the studies reported on the efficacy of vitamin D supplementation in dry eye disease (eight studies) [43, 49, 54, 57, 58, 60–62]. There were three studies of vitamin A [48, 53, 59] and two reports on vitamin B1 and B12 [50, 51]. No studies explored vitamin C oral supplementation in dry eye disease participants. Studies were conducted in nine countries, with one study each from Iran [61], Turkey [54], Pakistan [58], Saudi Arabia [53], South Korea [57] and Australia [43]; two studies each from Egypt [60, 62] and India [48, 59] and three studies from China [50, 51, 79]. The study designs were randomised clinical study (*n* = 6) [43, 48, 50, 51, 61, 79], pre-post intervention (*n* = 3) [53, 57, 60], cross-sectional (*n* = 2) [54, 59] and non-randomised clinical trials (*n* = 2) [58, 62]. Overall, the outcome parameters of the 13 included studies reported changes after vitamin supplementation in 483 participants, with individual study sample sizes ranging from 9 to 94 individuals. The unit of analysis was a single eye in four studies [51, 53, 54, 61], both eyes in three studies [48, 50, 60] and was unclear in six studies [43, 57–59, 62, 79]. Follow-up duration ranged from 3 days to 6 months. TBUT was performed in all the studies. The SH was reported in 10 studies [43, 48, 49, 54, 57–62], OSDI in 10 studies [43, 48, 49, 51, 54, 57–60, 62], CFSS in eight [43, 48, 49, 51, 54, 57, 59, 62], lid hyperaemia in three [54, 57, 62] and VAS in two studies [57, 62]. All the data were reported as mean ± SD, except for one study [59] where median and grades were reported for TBUT and SH. A control group was not included in seven studies [43, 49, 53, 54, 59, 60, 62].

### Methodological Quality Assessment

**Fig. 2:**
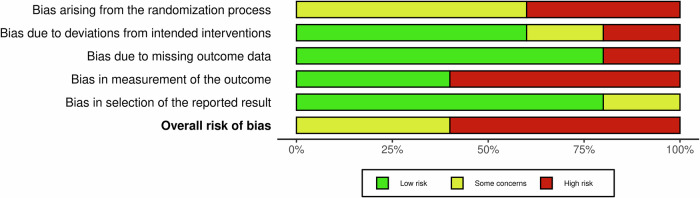
Weighted bar plots of risk of bias assessment for the included randomised clinical trials.

**Fig. 3:**
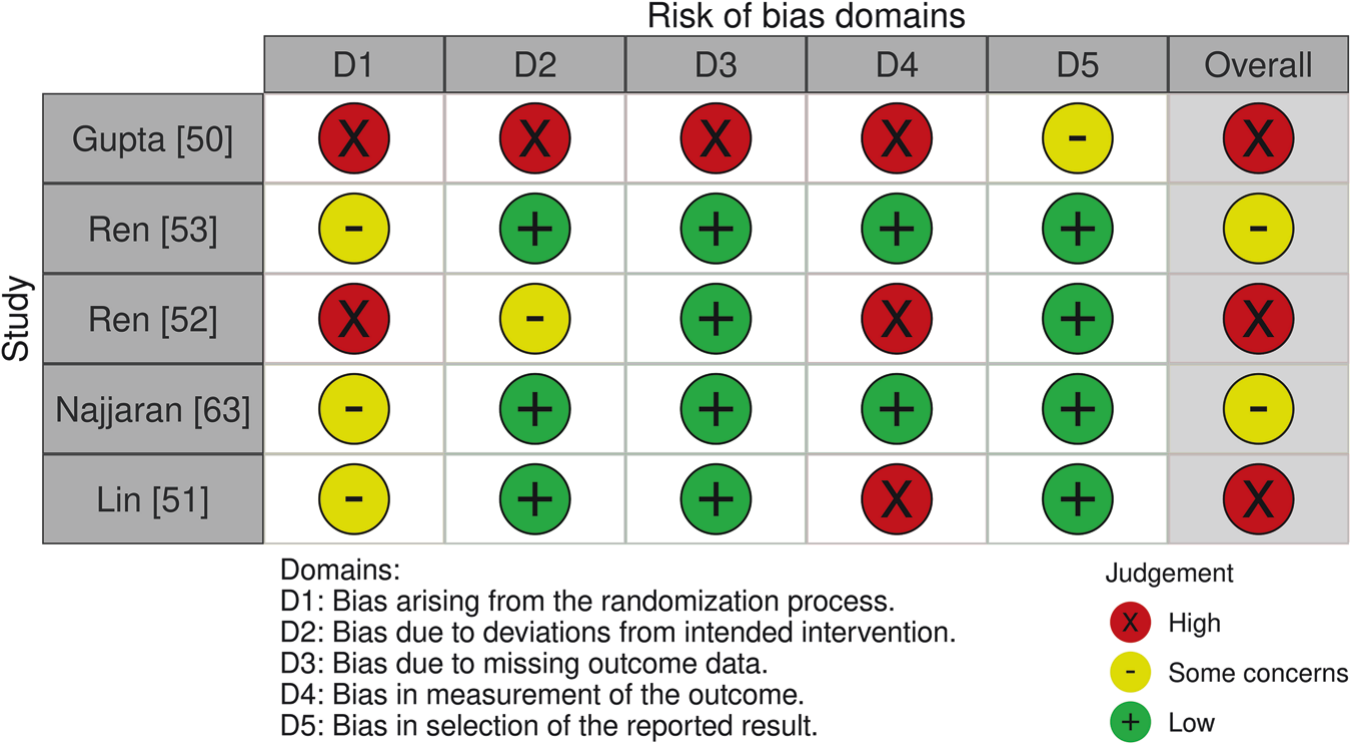
Traffic light plots showing risk of bias assessment for the included randomised clinical trials.

Figures 2 and 3 and Table 2 illustrate the details of quality assessment for risk of bias. Because the randomisation procedure was not disclosed, three [48, 50, 79] of the five RCTs [48–51, 61] were deemed to have a high risk of bias. In all of the RCTs, the randomisation process was not described properly, despite using randomised sequencing in grouping the study cases [48–51, 61]. As mentioned previously, the RoB 2 assessment of bias classifies the overall results into three different scores: low risk, some concerns and high risk of bias. Subsequently, two studies showed some concerns in the randomisation process based on the RoB2 results [51, 61].

The six case-control studies were judged to be of poor quality/high risk of bias [43, 57, 58, 60, 62] or fair [53] due to not defining the study population clearly, the absence of sample size calculations and insufficient information related to the ascertainment of the exposure and non-response rate. The two cross-sectional studies were judged to be of unsatisfactory quality [59] or fair [54] due to a lack of representativeness of the study participants, inadequate/absence of information on sample size calculations, ascertainment of the exposure and potential biases in the selection of the reported results.

### Extracted Data

Table 3 presents the mean ± SD of outcome measures for tear breakup time (TBUT), SH and ocular surface disease index (OSDI) in the randomised clinical trials (RCTs).

**Table 3:** Outcomes of randomised clinical trials.

First Author/Year	Vitamin type	SH Test (mm) Mean ± SD Before	SH Test (mm) Mean ± SD After	TBUT (s) Mean ± SD Before	TBUT (s) Mean ± SD After (follow-up days)	OSDI Mean ± SD Before	OSDI Mean ± SD After (follow-up days)
Najjaran 2022 [62]	Vit D	10.2 ± 4.5	12.5 ± 4.2	6.6 ± 1.4	10.5 ± 1.5	–	–
Lin 2022[50]	Vit D	7.2 ± 0.3	9.3 ± 0.4 (30) 11.3 ± 0.3 (90)	7.5 ± 1.4	10.7 ± 1.0 (30) 10.7 ± 1.0 (90) 10.7 ± 1.0 (180)	26.5 ± 8.3	11.6 ± 8.5 (30) 10.2 ± 5.4 (90) 10.4 ± 5.2 (180)
Ren 2020 [51]	Vit B1 & Mec*	–	–	5.7 ± 1.7 ^a^ 5.0 ± 1.9 ^b^	6.4 ± 1.4 (30) ^a⸸^ 6.4 ± 2.3 (60) ^a⸸^ 5.9 ± 1.9 (30) ^b⸸^ 6.7 ± 2.7 (60) ^b⸸^	–	–
Ren 2022 [52]	Vit B1 & Mec*	–	–	5.3 ± 0.2	4.9 ± 0.2 (30) 5.6 ± 0.3 (90)	39.3 ± 2.4	33.9 ± 2.6 (30) 29.3 ± 2.8 (90)

Mec* Mecobalamin. *OSDI* ocular surface disease index, *SD* standard deviation, *SH* Schirmer test, *TBUT* tear breakup time, *Vit* vitamin.

^⸸^Ren et al. study [50] reported both tear film breakup time average (TBUTA) and TBUT first time (TBUTF); however, just TBUTA is considered here.

^a^Outcomes of participants in the oral vitamin B1, mecobalamin and artificial tears group.

^b^Outcomes of participants in oral vitamin B1, mecobalamin, corticosteroid eye drops and artificial tears.

### Effects of Interventions

#### Vitamin A Studies

Three studies investigated the effect of oral vitamin A on dry eye disease using randomised comparative clinical trial [48], cross-sectional [59] and case-control designs [53]. These studies had highly variable follow-up durations, including both short and long-term perspectives [48, 53, 59]. The control group in the case-control study comprised sex- and age-matched healthy participants [53], whereas Gupta et al. compared oral omega-3 fatty acids with oral vitamin A in a randomised trial of participants with dry eye [48]. Jadeja et al used a single-arm interventional study [59]. Across these three studies, a total of 172 participants were included, with individual study sample sizes ranging from 42 to 70 participants [48, 53, 59]. In two studies, dosages of 5000 and 200,000 International Units (IU) were reported [53, 59], while no dosage was reported in one study [48]. Vitamin A administration in Alanazi et al. was for 3 consecutive days and the tests were performed 24 h after the last administered dose [53], while in Jadeja et al., participants were given vitamin A capsules on days 0, 1, 14, 28 and follow-up visits were scheduled at 1 month and 3 months from the 28th day of vitamin A supplementation [59]. For Gupta et al., oral vitamin A antioxidant was given once daily for 6 months [48].

Two studies evaluated OSDI [48, 59]. Jadeja et al. showed significant improvement of OSDI from 22.8 ± 7.8 at presentation to 20.0 ± 7.5 and 17.1 ± 7.3 after 1 (*p* < 0.01) and 3 months (*p* < 0.01), respectively [59]. However, Gupta et al. did not report OSDI results, but mentioned an improvement in symptom scores across follow-up periods [48]. Two studies evaluated the SH [48, 59]. One was measured without anaesthesia [59], while this was not indicated in the other [48]. Jadeja et al. reported that the mean score of the SH I changed significantly from 2.14 ± 0.96 on presentation, to 1.73 ± 0.77 at 1 month and 1.61 ± 0.70 at 3 months (*p* < 0.0001) [59]. Gupta et al. did not report any statistical analysis for the observed improvement [48]. Two studies evaluated TBUT using fluorescein [48, 53], but Jadeja et al. did not describe the method used [59]. The impact of oral vitamin A supplementation on TBUT and tear film stability was mixed. Alanazi et al. reported no statistically significant difference in TBUT values (7.8 ± 3.3 and 8.8 ± 4.5 s pre- versus post-supplementation, respectively (*p* = 0.49)) [53]. In contrast, two other studies reported significant improvement in TBUT [48, 59]. Gupta et al. observed increases from 3.5-1.5 to 6.2–3.1 s following supplementation, reflecting improvements in tear film stability (*p* < 0.05) [48]. Similarly, Jadeja et al. reported improvement for TBUT grades (duration not reported) from 1.8 ± 0.9 on presentation to 1.2 ± 0.4 at 3 months post-supplementation (*p* < 0.0001) [59].

#### Vitamin B1 and B12 Studies

Two RCTs by the same authors investigated the effect of oral vitamin B12, in combination with vitamin B1, on dry eye disease over two different time intervals [50, 51]. Both studies focused on symptom relief, sign improvement and patient satisfaction after supplementation. They also examined changes in corneal nerve parameters using in vivo confocal microscopy [50, 51]. Table 3 shows the TBUT measurements. Statistically significant improvement was shown in the 2020 study (*p* < 0.05) [50]. It should be noted that the significant changes in TBUT did not occur consistently across all follow-up visits, and significant differences were only found between baseline and after 2 months of follow-up. In contrast, the 2022 study reported no significant change in TBUT (*p* > 0.05) [51]. However, optimised OSDI scores changed significantly (*p* < 0.05) from 39.3 ± 2.4 at baseline to 33.9 ± 2.6 and 29.3 ± 2.8 after 1 and 3 months, respectively (*p* < 0.05). CFSS changed from 0.5 ± 0.1 at baseline to 0.2 ± 0.1 and 0.1 ± 0.1 after 1 and 3 months of oral supplementation, respectively (*p* < 0.05) [51]. The original studies reported statistical significance only as threshold values (*p* < 0.05) and did not provide exact *p*-values.

#### Vitamin D

Eight studies investigated the impact of oral vitamin D administration using RCTs, single-arm before–after intervention studies or cross-sectional study designs [43, 49, 54, 57, 58, 60–62]. Across these studies, a combined total of 304 participants were enroled; with individual study sample sizes ranging from 9 to 54 participants (Table 1) [43, 49, 54, 57, 58, 60–62]. The RCTs evaluated the outcome measures at baseline and at 56 days, 1 month and 3 months post-supplementation (Table 1) [49, 61]. The participants receiving vitamin D supplementation were compared with a control group, with follow-up durations ranging from 2 to 24 weeks after vitamin intake [43, 54, 57, 58, 60, 62]. These studies differed in terms of the TBUT, SH, lid hyperaemia and CFSS findings. Three studies reported no significant difference in TBUT after 14, 60 and 106 days (*p* > 0.05) [43, 57, 62]. They also observed no significant difference in SH scores when measured without anaesthesia. The remaining studies [49, 54, 58, 60, 61] noted significant differences in TBUT and SH scores post-vitamin D supplementation (*p* < 0.05) [49, 54, 58, 60]. However, one study [57] found that oral vitamin D supplementation did not significantly improve dry eye symptoms measured by OSDI, even though the average score decreased slightly (50.5 ± 15.9 and 43.2 ± 12.9 before and after oral cholecalciferol, respectively; *p* = 0.72) [57].

Fayed et al. reported a significant increase in OSDI scores among vitamin D-deficient participants, rising from 32.5 ± 9.6 at baseline to 42.3 ± 2.4 after 2 weeks, before decreasing to 35.3 ± 2.5 at 4 months [62]. These changes were significantly different from those observed in the non-vitamin D-deficient group (*p* < 0.05)[62]. Interestingly, when participants were able to select their source of vitamin D supplementation, no significant difference in CFSS measurements was found (pre- and post- supplementation values of 0.3 ± 0.8 and 0.0 ± 0.0, respectively; *p* > 0.99) [57]. Likewise, two studies [43, 62] reported no significant change in CFSS after treatment, Fayed et al.—baseline and post-treatment values of 0.5 ± 0.6 and 0.5 ± 0.7, *p* > 0.87, respectively; Yang et al.—baseline and post-treatment values of 0.5 ± 0.7 and 0.3 ± 0.5, *p* > 0.99, respectively [43, 62]. Based on the data from these two separate studies with similar designs [57, 62], lid hyperaemia and VAS did not differ significantly following therapy (*p* > 0.05).

## Discussion

This systematic review investigated the effects of oral vitamin supplementation on dry eye disease. Of the 13 eligible studies, most investigated the efficacy of vitamin D supplementation (8 studies; 61.5%), followed by vitamin A (3 studies; 23.1%) and a combination of vitamin B1 and mecobalamin (2 studies; 15.4%). Notably, no clinical studies evaluating the effects of vitamin C supplementation on dry eye disease met the inclusion criteria. The growing interest in vitamin D highlights its immunomodulatory role in dry eye disease, whereas evidence to support the use of other vitamins remains limited. Based on the risk of bias assessments, none of the studies were judged to be of high quality or exhibit minimal risk of bias. The main reasons for downgrading the quality assessment were the failure to mask the investigators, explain the randomisation process, include an appropriate control group or report sample size calculations. This review identified only four relevant studies with an RCT design (25%); most of the included investigations used a pre–post intervention design. According to the National Health and Medical Research Council of Australia’s evidence hierarchy, studies using pre-post designs provide a relatively low degree of empirical evidence for intervention questions (Level IV) [80]; this recognises the inherent limitations and bias risk in these designs. These limitations should be considered during the interpretation of the results. Thus, future research should consider the advantages of an RCT design, where feasible, in order to reduce bias and build a body of evidence. This will enable future studies to achieve more certainty regarding the impact of oral vitamin supplementation on tear film and ocular surface parameters. Of the 13 included studies, none reported any adverse effects. Therefore, investigations into the safety of oral vitamin administration on dry eye and the ocular surface are limited and a risk-benefit analysis cannot be conducted. It is suggested to assess and report adverse events in future studies.

### Vitamin A

Two of the three studies [48, 59] conducted on vitamin A were in agreement regarding a positive effect on OSDI, but not in agreement regarding both the SH and TBUT. Vitamin A is crucial for maintaining the health and function of the goblet cells, responsible for mucin production, which improves corneal surface wettability [81]. Therefore, vitamin A deficiency can lead to xerophthalmia, which is a consequence of various ocular surface disorders [82], highlighting the importance of adequate intake through diet and supplementation. An improvement in OSDI with vitamin A supplementation might be due to enhanced mucin production or an improvement in ocular surface integrity as a result of regulation of corneal epithelial cell proliferation and differentiation [23, 83]. Importantly, clinical signs may take up to 4 months to show improvement [11] and one study just had a 3-day follow-up period [53]. However, only Jadeja et al. reported OSDI results [59], while Gupta et al. mentioned an improvement without reporting the results [48]. This shows a serious risk of bias in the reported results. Other reasons for differences in findings might be attributed to different study protocols, the diversity of administered dosages and formulations, dissimilar durations of intake, characteristics of the study population, variability in the severity of dry eye disease at each stage of the study, baseline vitamin A serum levels and other factors, including environmental conditions, variations in ethnicity and race and concomitant medications. It is important to distinguish between preformed vitamin A (retinol and retinyl esters) versus pro-vitamin A carotenoids, as these chemical forms differ substantially in bioavailability, metabolism and toxicity profiles. Acute vitamin A toxicity is primarily associated with excessive short-term consumption, typically >100,000 retinol activity equivalents (RAE) [84]. Although acute vitamin A toxicity is rare, it is more likely to occur with the administration of synthetic forms, including retinoid medication and isotretinoin, due to their direct biological activity and potential for accumulation [84]. On the other hand, chronic vitamin A toxicity typically occurs with excessive consumption of preformed vitamin A > 8000 RAE per day, most commonly from animal-based foods rich in retinol such as liver, or high-dose supplements [85]. In contrast, pro-vitamin A carotenoids, such as β-carotene, require enzymatic conversion to retinol, a process that is tightly regulated according to the physiological need; thereby reducing the risk of classical hypervitaminosis A significantly [86]. The major adverse effect of retinoids, related to the ocular surface, is meibomian gland dysfunction, resulting in atrophy of acini and hyposecretion of oils [87, 88]. However, this side effect is not reported to be dose-related and is reversible upon discontinuation of the drug [87]. Therefore, eye care practitioners are advised to be mindful when suggesting vitamin A supplementation for dry eye patients because it could worsen their condition. Unfortunately, across the studies included here, the specific chemical form of vitamin A was reported inconsistently, limiting the ability to differentiate outcomes by provitamin A carotenoids versus preformed retinoids, in order to interpret safety and toxicity with high precision. Further well-designed clinical studies employing clearly defined chemical forms of vitamin A, with larger sample sizes, more standardised dosages and durations, and more controlled confounding factors are required to provide definite answers regarding the safety and therapeutic role of vitamin A supplementation in dry eye disease.

### Vitamin B1 and B12

The two studies conducted on vitamin B1 and mecobalamin (an endogenous form of vitamin B12) by the same research group are in agreement regarding an improvement in dry eye disease symptoms [50, 51], although the OSDI was used in only one of the studies as a measurement parameter [51]. The findings of two studies regarding TBUT results were inconsistent. An earlier study reported significant improvement in TBUT following supplementation after 2 months of treatment [50]. In contrast, the later investigation did not demonstrate a statistically significant change in TBUT, but did observe an improvement in CFSS [51]. Vitamins B1 and B12 are known for their neuroprotective and anti-inflammatory effects, which may reduce neuropathic ocular pain or ocular surface sensitivity, even if tear film stability does not change significantly [76, 78]. The analgesic or antinociceptive impact of vitamin B has been shown in in vivo studies [89]. These investigations have reported enhanced inhibitory regulation of afferent nociceptive neurons in the spinal cord, reduced response of thalamic neurons to nociceptive stimuli, involvement of serotonergic pathways in pain modulation or activated opioid receptors [90, 91]. Consequently, these functions could be a potential reason for improved OSDI scores. Therefore, improved CFSS results could be due to enhanced nerve repair and facilitated cellular energy production by vitamin B12, and as a result, reduced apoptosis of epithelial cells [92].

The contrasting findings between the two studies are likely due to differences in the participating treatment groups. In 2020, Ren et al. recruited patients with dry eye disease, subbasal nerve layer injury and the presence of dendritiform cells [50]. It has been reported that neurosensory dysfunction is more likely to present in severe dry eye [93]. Therefore, one reason for the improved OSDI scores observed in this specific investigation might be due to a more dysfunctional corneal nerve layer at baseline, compared with the later study [51]. Unfortunately, the exact definition of subbasal nerve layer injury was not provided in the first study [50]. Still, based on the improvement in corneal nerve fibre density after treatment, it suggests that the injury included a reduction in corneal nerve layer density. In other words, activated dendritic cells and subbasal dendritic cells in the corneal nerve layer both suggest an ongoing inflammatory process as an essential component of the immune response in dry eye disease [9, 94, 95]. Therefore, due to indirect axonal flow enhancement, the contribution to acetylcholine synthesis by thiamine and the anti-inflammatory and myelin sheath maintenance induced by cobalamin properties, better results were anticipated in patients with a more severely damaged corneal nerve layer compared to those with just dry eye disease [96–99]. A second article by the same authors used a larger sample size based on the TFOS Dry Eye Workshop II (DEWS II) criteria [1], without specifically targeting those with corneal nerve layer damage [51]. Other factors that might have affected the effective outcomes of these studies included a smaller sample size, shorter follow-up duration, differences in control groups and study design. However, given the analgesic effect of the vitamin B1-cobalamin combination, this remains a promising therapeutic option for improving patient outcomes in dry eye disease. In general, B vitamins are essential for the normal functioning of the nervous system [100]. However, in rare cases, adverse effects have been associated with excessive administration of vitamins B1 and B12 [101, 102]. There are no reports regarding the safety of oral thiamine consumption. In contrast, the parenteral route in higher doses has been associated with phlebitis, and rarely sensitivity reactions such as nausea and indigestion [103, 104]. Interestingly, an oral dose of 3 g/day or higher over long-term periods does not seem to have deleterious effects [105]. Dermal changes and acne have been associated with vitamin B12 administration in rare cases, and the risk appears to be higher with hydroxycobalamin than with cyanocobalamin [106]. These adverse reactions were mainly reported with high doses administered parenterally [106]. To the contrary, it has also been suggested that high-dose, long-term oral vitamin B12 has no side effects [107]. Nevertheless, practitioners should always consider potential side effects, and an individual approach should be taken. This might include checking serum blood levels, regular follow-up visits or referral to a general medical practitioner. Further research is necessary to evaluate the mechanisms involved in the inconsistent TBUT findings, including determining the optimal dosage and frequency of supplementation, to enhance an understanding of the therapeutic potential of vitamins B1 and B12.

### Vitamin D

Eight studies were conducted on vitamin D [43, 49, 54, 57, 58, 60–62] and seven of them used OSDI as an evaluation tool. Six of these studies were in agreement regarding a positive effect on the OSDI [43, 49, 54, 58, 60, 62], whereas the TBUT, SH, lid hyperaemia and CFSS findings were not in agreement [43, 57, 62]. Supplementation with vitamin D significantly reduced the OSDI score [43, 49, 54, 58, 60–62]. The proposed mechanisms for a beneficial role of vitamin D on ocular surface health frequently involved its anti-inflammatory and immunomodulatory properties (109). There is a body of evidence that vitamin D deficiency may increase the risk of squamous metaplasia and ocular surface goblet cell loss (33), consequently reducing tear film stability and quality, as well as ocular surface wettability (33). Inflammation is one of the main mechanisms underlying dry eye disease and vitamin D promotes surfactant release and phospholipid synthesis [108]. The phospholipids form a thin polar layer which contributes to tear viscosity, while surfactants reduce surface tension and regulate inflammation [109, 110]. Accordingly, vitamin D may reduce the OSDI through increased surfactant and phospholipid secretion. The absence of a significant change in the OSDI in Hwang et al. may be due to the short follow-up duration of only 2 weeks and an extremely small sample size (*n* = 9), compared with other studies [57]. A longer supplementation period should be tested. Furthermore, the participants in this study were able to select the route of treatment, either oral or intramuscular, potentially biasing the results [57]. Drawbacks in the design of this study, as well as the lack of compliance monitoring, make it difficult to draw a conclusion about the efficacy of oral vitamin D supplementation.

In addition, Yang et al. [43] reported no significant improvement in either tear stability or SH results. This may have been due to high baseline NIBUT and SH findings. Pre- and post-supplementation TBUT findings were 12.2 ± 6.7 and 10.3 ± 5.4 s, respectively (adjusted *p* > 0.99). Respective results for the SH were 22.0 ± 10.8 and 21.0 ± 12.5 (adjusted *p* > 0.99). Lower tear secretion in persons with vitamin D deficiency might be due to reduced parasympathetic function, which leads to reduced tear release from the lacrimal gland, which is innervated primarily by parasympathetic nerve fibres [111, 112]. As the parameters described fell within the normal range at baseline, suggesting normal parasympathetic innervation to the lacrimal glands, vitamin D may not improve efficacy further [43]. Notably, most studies integrated this treatment method with conventional topical applications such as artificial tears [49, 58, 61, 62]. This could be a confounding factor in investigating the impact of vitamin administration alone.

Three studies reported no significant improvement in CFSS [43, 57, 62]. A systematic review and meta-analysis showed that CFSS, as an outcome parameter for corneal epithelial cell damage, reflects severe but not mild or moderate dry eye disease [113]. The lack of a significant change in CFSS is likely because of the lower CFSS scores at baseline or the presence of only mild or moderate dry eye in the study participants. Similarly, mild baseline VAS scores might reduce the chance for measurable improvement. Furthermore, the subjective nature of the test and individual ratings could also account for the non-significant findings.

One possible explanation for the inconsistency of the measured findings across the studies included could be attributed to differences in the severity of dry eye disease in each participant group. The absence of a baseline serum vitamin D evaluation before starting therapy is a significant limitation in the context of vitamin D supplementation. Additionally, individual differences in outdoor activity and exposure to ultraviolet (UV) radiation, which have a significant effect on endogenous vitamin D production [114], were rarely documented or considered. Other factors, including different metabolite choices, dosages, short follow-up times and small sample sizes, may have also contributed to these discrepancies.

Hypervitaminosis D or vitamin D toxicity is a rare but potentially serious condition that occurs in the presence of excessive vitamin D in the body [115]. High blood calcium concentrations can cause over-calcification of the bones, soft tissues, heart and kidneys [115]. Hypercalcemia is a condition caused by elevated calcium levels secondary to excessively high levels of vitamin D. The side effects of vitamin D treatment include increased thirst, a metallic taste in the mouth, stomach symptoms and excessive fatigue [116]. Therefore, it is suggested that practitioners strive to evaluate vitamin D serum levels optimally in cases of severe dry eye disease.

To build upon the current literature, future studies should reduce the possible risk of bias by using RCT designs. Clinicians should be advised to check vitamin D blood serum levels in cases of treatment-refractory dry eye disease patients. Vitamin D supplementation could be a potential adjuvant therapy to conventional artificial tears in ocular surface disease. However, inappropriate overprescribing of vitamin D supplements can lead to toxicity and life-threatening hypercalcemia [117, 118]. While vitamin D toxicity is extremely rare, it can occur with the administration of excessively high doses [117, 118].

## Conclusion and New Frontiers

This report explored the available clinical evidence on oral vitamin supplementation for dry eye disease. The current review, which included only published studies from 2017 to 2023, does not offer sufficient high-quality evidence to confirm the effectiveness of vitamin supplementation in the treatment of dry eye disease, despite the biological potential and mechanistic rationale suggested by experimental and observational research. Importantly, none of the included studies was judged to be of high methodological quality or at minimal risk of bias and most were limited by small sample sizes, short follow-up durations, heterogenous outcome measures and inadequate control of confounding factors.

Consequently, based on the existing evidence, the null hypothesis must be accepted, indicating that there is insufficient scientific support to recommend oral vitamin supplementation as an effective treatment for dry eye disease. Although it has been suggested that vitamins A, D and B complex affect ocular surface physiology through roles in immune modulation [27–30], neural function [50, 51] and epithelial integrity [23, 83], these mechanistic considerations are not adequate to support their clinical use in the absence of solid, well-controlled trials.

Therefore, adequately powered RCTs with precise vitamin formulations, baseline nutritional evaluations, standardised outcome measures, and appropriate control groups should be a priority for future research. The existing literature does not support routine vitamin supplementation for dry eye disease.

## Supplementary information

Supplementary information

## Data Availability

Data described in the manuscript, code book and analytic code will be made available upon request, pending.
