# Correction: The Efficacy of Oral Vitamin Supplementation in Dry Eye Disease: A Systematic Review

**DOI:** 10.1007/s44402-026-00116-1

**Published:** 2026-06-16

**Authors:** Hamidreza Heidari, Maria Markoulli, Jayashree Arcot, Asgar Doostdar, Azadeh Tavakoli

**Affiliations:** 1 School of Optometry and Vision Science, Faculty of Medicine, UNSW Sydney, Kensington, New South Wales Australia; 2 Food and Health group, School of Chemical Engineering, Faculty of Engineering, UNSW Sydney, Kensington, New South Wales Australia; 3 Department of Optometry, School of Rehabilitation Sciences, Iran University of Medical Sciences, Tehran, Iran

Correction to: *Ophthalmic and Physiological Optics* 10.1007/s44402-026-00079-3, published online 26 April 2026

In this article, the author's name, ‘Azadeh Tavakoli’ has been erroneously published as ‘Azadeh Tavakkolinasab’.

The original article has been updated.

