# Supplementary material for: The Efficacy of Oral Vitamin Supplementation in Dry Eye Disease: A Systematic Review
Source: Ophthalmic Physiol Opt. 2026 Apr 15;46(3):568–79. doi: 10.1007/s44402-026-00079-3 (PMC13369572; doi:10.1007/s44402-026-00079-3)
Supplement: Supplementary file 1 — Supplementary information [file 44402_2026_79_MOESM1_ESM.docx]

**Table S1:** Reasons for excluding studies at the full-text screening stage

| **Study identification** | **Inclusion criteria not met** |
| --- | --- |
| Djunaedi et al. (1988) (1) | Study outcomes |
| Dutta et al. (2014) (2) | study population |
| Fukuoka et al. (2021) (3) | study design |
| Galor et al. (2014) (4) | intervention |
| Iwuagwu et al. (2003) (5) | study population |
| Katz et al. (1995) (6) | outcomes |
| Kim et al. (2024) (7) | outcomes |
| Kizilgul et al. (2018) (8) | route of administration |
| Kurtul et al. (2015) (9) | study design |
| Ozen et al. (2017) (10) | route of administration |
| Peponis et al. (2002) (11) | study population |
| Peponis et al. (2004) (12) | study population |
| Shetty et al. (2016) (13) | route of administration |
| Shetty et al. (2015) (14) | route of administration |
| Singman et al. (2013) (15) | outcomes |
| Sullivan et al. (1973) (16) | study population |
| van Agtmaal et al. (1988) (17) | outcomes |
| Vetrugno et al. (2001) (18) | outcomes |
| Watts et al. (2020) (19) | route of administration |
| Ziada et al. (2004) (20) | study design |

**Table S2:** **Assessment of risk of bias in included clinical trial studies, for each domain in the RoB 2 tool.**

| **Study** | **Domain 1** | | | **Domain 2** | | | | | | | **Domain 3** | | | | **Domain 4** | | | | | **Domain 5** | | | **Overall risk of Bias** |
| --- | --- | --- | --- | --- | --- | --- | --- | --- | --- | --- | --- | --- | --- | --- | --- | --- | --- | --- | --- | --- | --- | --- | --- |
|  | **1.1** | **1.2** | **1.3** | **2.1** | **2.2** | **2.3** | **2.4** | **2.5** | **2.6** | **2.7** | **3.1** | **3.2** | **3.3** | **3.4** | **4.1** | **4.2** | **4.3** | **4.4** | **4.5** | **5.1** | **5.2** | **5.3** |  |
| Gupta et al. (2021) (21) | PY^a^ | NI^b^ | PY | PY | PY | NI | NA^c^ | NA | PN^d^ | NI | NI | PN | NI | NI | PN | PN | PY | PY | PY | PN | PN | PN | High |
| Ren et al. (2020) (22) | PY | PN | PY | PY | PY | NI | NA | NA | PY | NA | PY | NA | NA | NA | PN | PN | PY | NI | NI | Y^e^ | PN | PN | High |
| Ren et al. (2022) (23) | Y | PY | PN | N^f^ | N | NA | NA | NA | Y | NA | Y | NA | NA | NA | PN | PN | PN | NA | NA | Y | PN | PN | Low |
| Najjaran et al. (2023) (24) | PY | NI | PN | PY | PY | PN | NA | NA | PY | NA | PY | NA | NA | NA | PN | PN | PY | PN | NA | Y | PN | PN | Some concerns |
| Lin et al. (2022)(25) | PY | NI | PN | PY | PY | PN | NA | NA | PY | NA | NI | PN | PN | NA | PN | PN | PY | PY | PY | Y | PN | PN | High |

a: Probably Yes; b: No Information; c: Not Applicable; d: Probably No; e: Yes; f: No.

**List of domains**

1.1- Was the allocation sequence random?

1.2- Was the allocation sequence concealed until participants were enrolled and assigned to interventions?

1.3-Did baseline differences between intervention groups suggest a problem with the randomization process?

2.1- Were participants aware of their assigned intervention during the trial?

2.2- Were carers and people delivering the interventions aware of participants' assigned intervention during the trial?

2.3- If Y/PY/NI to 2.1 or 2.2: Were there deviations from the intended intervention that arose because of the trial context?

2.4- Were these deviations likely to have affected the outcome?

2.5- Were these deviations from intended intervention balanced between groups?

2.6- Was an appropriate analysis used to estimate the effect of assignment to intervention?

3.1- Were data for this outcome available for all, or nearly all, participants randomized?

3.2- If N/PN/NI to 3.1: Is there evidence that the result was not biased by missing outcome data?

3.4- If N/PN to 3.2: Could missingness in the outcome depend on its true value?

3.5- If Y/PY/NI to 3.3: Is it likely that missingness in the outcome depended on its true value?

4.1- Was the method of measuring the outcome inappropriate?

4.2- Could measurement or ascertainment of the outcome have differed between intervention groups?

4.3- If N/PN/NI to 4.1 and 4.2: Were outcome assessors aware of the intervention received by study participants?

4.4- If Y/PY/NI to 4.3: Could assessment of the outcome have been influenced by knowledge of intervention received?

4.5- If Y/PY/NI to 4.4: Is it likely that assessment of the outcome was influenced by knowledge of intervention received?

5.1- Were the data that produced this result analysed in accordance with a pre-specified analysis plan that was finalized before unblinded outcome data were available for analysis?

5.2- ... multiple eligible outcome measurements (e.g. scales, definitions, time points) within the outcome domain?

5.3- ... multiple eligible analyses of the data?

**Appendix SI:** Search strategies

**PubMed**

1. (vitamin[all] OR vitamins[all] OR vitamin*[all] OR vitamin B12*[all] OR vitamin D[all] OR cynacobalamin[all] OR cobalamin*[all] OR eritron[all] OR vitamin A*[all] OR vitamin C*[all] OR brow[all] OR canthus[all] OR canthal[all] OR conjunctiv*[all] OR cornea*[all] OR ascorbic acid*[all] OR eyel*[all] OR “eye lid*”[all] OR “eye lash*”[all] OR episcler*[all] OR lacrima*[all] OR goblet cell*[all] OR “lid wiper”[all] OR meibomi*[all] OR tear[all] OR tears[all])
2. (“vitamins”[mesh] OR “vitamin D”[mesh] OR “vitamin D/therapeutic use”[mesh] OR “vitamin B12”[mesh] OR “vitamin B12/therapeutic use”[mesh] OR “ascorbic acid”[mesh] OR “ascorbic acid/therapeutic use”[mesh] OR “vitamin A”[mesh] OR “vitamin A/therapeutic use”[mesh] OR "Dietary Supplements"[Mesh]) OR "Nutrition Therapy"[Mesh])
3. #1 OR #2
4. (“ocular surface cell*”[all] OR “dry eye disease*”[all] OR “severe dry eye disease”[all] OR “ded”[all] OR “dry eye syndrome”[all] OR “dry eye*”[all] OR “evaporative dry eye disease”[all] OR “evaporative dry eye*”[all] OR “evaporative dry eye syndrome”[all] OR “meibomian gland dysfunction*”[all] OR “cornea”[all] OR “Tears”[all])
5. "Dry Eye Syndromes"[MeSH Terms] OR "Meibomian Gland Dysfunction"[MeSH Terms] OR "Meibomian Glands"[MeSH Terms] OR "Lacrimal Apparatus"[MeSH Terms] OR "epithelium, corneal"[MeSH Terms] OR "Tears"[MeSH Terms] OR "Conjunctivitis"[MeSH Terms]
6. #4 OR #5
7. (“effect*”[all] OR “treatment*”[all] OR “drug therapy”[all] OR “impact”[all] OR “supplement”[all] OR “ocular absorption”[all] OR “therapeuthics*”[all] OR “supplement*”[all] OR “Diet therapy”[all] OR “administration”[all] OR “Oral administration”[all] OR “treatment outcome”[all])
8. ("Ocular Absorption"[MeSH Terms] OR "Therapeutics"[MeSH Terms] OR "Drug Therapy"[MeSH Terms] OR "Diet Therapy"[MeSH Terms] OR "administration, ophthalmic"[MeSH Terms] OR "administration, oral"[MeSH Terms] OR "administration, topical"[MeSH Terms] OR "Treatment Outcome"[MeSH Terms:noexp])
9. #7 OR #8
10. #3 AND #6 AND #9

**Embase**

1. ocular surface disease/ or tear film/ or eyelid/ or cornea/ or cornea disease/ or cornea endothelium/ or cornea epithelium/ or cornea limbus/ or corneal neovascularization/ or cornea opacity/ or cornea stroma/ or cornea thickness/ or cornea tissue/ or anterior eye segment/ or canthus/ or conjunctiva/ or lacrimal apparatus/

2. exp dry eye/

3. (vision or sight* or ocular or occular or limbus or limbal or orbit* or blink* or canthus or canthal or conjunctiv* or cornea* or corneo* or eyel* or eye-lid* or eye-lash* or episcler* or lacrima* or goblet-cell* or lid-wiper or meibomi* or tear or tears).ab,ti.

4. exp "Vitamin D"/ or exp "Vitamin B 12"/ or exp "Ascorbic Acid"/ or exp "Vitamin A"/

5. treatment outcome/ or clinical outcome/ or clinical significance

6. 1 or 2 or 3

7. 4 and 5 and 6

**CINAHL**

1. MH "Vitamin D+" OR MH "Vitamin B 12+" OR MH "Ascorbic Acid+" OR MH "Vitamin A
2. MH "Dry Eye Syndromes+”
3. MH “Treatment Outcome” or MW “Drug Therapy”
4. 1 and 2 and 3

**Google Scholar**

("dry eye" OR "dry eye disease" OR "ocular surface disease" OR "DED") AND ("oral vitamin" OR "vitamin supplementation" OR "nutritional supplements" OR "dietary supplements" OR "micronutrients") AND ("impact" OR "effect" OR "efficacy" OR "influence" OR "role" OR "benefit" OR "therapeutic effect" OR "treatment outcome" OR "clinical outcome" OR "improvement") AND ("Schirmer test" OR "tear break-up time" OR "TBUT" OR "fluorescein staining" OR "conjunctival staining" OR "lid hyperemia" OR "ocular symptoms" OR "OSDI" OR "VAS")

References

1. Djunaedi E, Sommer A, Pandji A, Kusdiono, Taylor HR. Impact of vitamin A supplementation on xerophthalmia. A randomized controlled community trial. Archives of ophthalmology (Chicago, Ill : 1960). 1988;106(2):218-22.

2. Dutta S, Islam MN, Chakroborty S, Mondal A, Bandopadhay R, Gayen S, et al. Effect of anti-oxidant on tear film in patients suffering from diabetes mellitus. Journal of the Indian Medical Association. 2014;112(2):108-9.

3. Fukuoka S, Arita R, Mizoguchi T, Kawashima M, Koh S, Shirakawa R, et al. Relation of Dietary Fatty Acids and Vitamin D to the Prevalence of Meibomian Gland Dysfunction in Japanese Adults: The Hirado-Takushima Study. J Clin Med. 2021;10(2):16.

4. Galor A, Gardener H, Pouyeh B, Feuer W, Florez H. Effect of a Mediterranean Dietary Pattern and Vitamin D Levels on Dry Eye Syndrome. Cornea. 2014;33(5):437-41.

5. Iwuagwu F, Agu G, Azuamah Y, Okolie VJJotNOA. The effects of Vitamin A on tear break-up time of young adults. 2003;10.

6. Katz J, West K, Khatry SK, Thapa M, LeClerq SC, Pradhan E, et al. Impact of vitamin A supplementation on prevalence and incidence of xerophthalmia in Nepal. 1995;36(13):2577-83.

7. Kim JM, Choi YJ. Impact of Dietary Nutrients on the Prevalence of Dry Eye Syndrome among Korean Women Aged 40 and above: Evidence from the Korea National Health and Nutrition Examination Survey. Nutrients. 2024;16(3).

8. Kizilgul M, Kan S, Ozcelik O, Beysel S, Apaydin M, Ucan B, et al. Vitamin D Replacement Improves Tear Osmolarity in Patients with Vitamin D Deficiency. Semin Ophthalmol. 2018;33(5):589-94.

9. Kurtul B, Özer P, Aydinli MJE. The association of vitamin D deficiency with tear break-up time and Schirmer testing in non-Sjögren dry eye. 2015;29(8):1081-4.

10. Ozen S, Ozer MA, Akdemir MO. Vitamin B12 deficiency evaluation and treatment in severe dry eye disease with neuropathic ocular pain. Graefes Arch Clin Exp Ophthalmol. 2017;255(6):1173-7.

11. Peponis V, Papathanasiou M, Kapranou A, Magkou C, Tyligada A, Melidonis A, et al. Protective role of oral antioxidant supplementation in ocular surface of diabetic patients. Br J Ophthalmol. 2002;86(12):1369-73.

12. Peponis V, Bonovas S, Kapranou A, Peponi E, Filioussi K, Magkou C, et al. Conjunctival and tear film changes after vitamin C and E administration in non-insulin dependent diabetes mellitus. Medical science monitor : international medical journal of experimental and clinical research. 2004;10(5):Cr213-7.

13. Shetty R, Deshpande K, Deshmukh R, Jayadev C, Shroff R. Bowman Break and Subbasal Nerve Plexus Changes in a Patient With Dry Eye Presenting With Chronic Ocular Pain and Vitamin D Deficiency. Cornea. 2016;35(5):688-91.

14. Shetty R, Deshpande K, Ghosh A, Sethu S. Management of Ocular Neuropathic Pain With Vitamin B12 Supplements: A Case Report. Cornea. 2015;34(10):1324-5.

15. Singman EL, Poon D, Jun AS. Putative corneal neuralgia responding to vitamin D supplementation. Case Rep Ophthalmol. 2013;4(3):105-8.

16. Sullivan WR, McCulley JP, Dohlman CH. Return of goblet cells after vitamin A therapy in xerosis of the conjunctiva. American journal of ophthalmology. 1973;75(4):720-5.

17. van Agtmaal EJ, Bloem MW, Speek AJ, Saowakontha S, Schreurs HP, van Haeringen NJ. The effect of vitamin A supplementation on tear fluid retinol levels of marginally nourished preschool children. Curr Eye Res. 1988;7(1):43-8.

18. Vetrugno M, Maino A, Cardia G, Quaranta GM, Cardia L. A randomised, double masked, clinical trial of high dose vitamin A and vitamin E supplementation after photorefractive keratectomy. The British journal of ophthalmology. 2001;85(5):537-9.

19. Watts P, Sahai A, Kumar PR, Shamshad MA, Trivedi GK, Tyagi L. A prospective study to assess the role of vitamin D individually and in combination with cyclosporine in the treatment of dry eye in patients with deficient serum 25(OH)D levels. Indian journal of ophthalmology. 2020;68(6):1020-+.

20. Ziada HEAJIJoOR. Oral versus topical vitamin A antioxidant in treatment of dry eye syndrome. 2017;3(4):252-8.

21. Gupta A, Gahlot A, Singh D, Sharma AJEB. A Prospective and Comparitive Study of Role of Oral Omega 3 Versus Vitamin a Antioxidant in Treatment of Dry Eye Syndrome. 2021;1:63-8.

22. Ren X, Chou Y, Jiang X, Hao R, Wang Y, Chen Y, et al. Effects of Oral Vitamin B1 and Mecobalamin on Dry Eye Disease. J Ophthalmol. 2020;2020:9539674.

23. Ren X, Chou Y, Wang Y, Jing D, Chen Y, Li X. The Utility of Oral Vitamin B1 and Mecobalamin to Improve Corneal Nerves in Dry Eye Disease: An In Vivo Confocal Microscopy Study. Nutrients. 2022;14(18).

24. Najjaran M, Zarei- Ghanavati S, Arjmand Askari E, Eslampoor A, Ziaei M. Effect of oral vitamin D supplementation on dry eye disease patients with vitamin D deficiency. Clin Exp Optom. 2023;106(3):257-62.

25. Lin Y, Su H, Wu J, Yuan M, Zhang YJIO. Oral vitamin D3 supplementation for femtosecond LASIK-associated dry eye vitamin D for LASIK dry eye syndrome. 2022;42(10):3145-52.
